# Significantly enhancing recombinant alkaline amylase production in *Bacillus subtilis* by integration of a novel mutagenesis-screening strategy with systems-level fermentation optimization

**DOI:** 10.1186/s13036-016-0035-2

**Published:** 2016-10-17

**Authors:** Yingfang Ma, Wei Shen, Xianzhong Chen, Long Liu, Zhemin Zhou, Fei Xu, Haiquan Yang

**Affiliations:** 1 The Key Laboratory of Industrial Biotechnology, Ministry of Education, School of Biotechnology, Jiangnan University, Wuxi, 214122 China; 2 The Key Laboratory of Carbohydrate Chemistry and Biotechnology, Ministry of Education, Jiangnan University, Wuxi, 214122 China

**Keywords:** *B. subtilis*, ARTP mutagenesis, Alkaline amylase, Fermentation optimization, Fed-batch, Overexpression

## Abstract

**Background:**

Alkaline amylase has significant potential for applications in the textile, paper and detergent industries, however, low yield of which cannot meet the requirement of industrial application. In this work, a novel ARTP mutagenesis-screening method and fermentation optimization strategies were used to significantly improve the expression level of recombinant alkaline amylase in *B. subtilis* 168.

**Results:**

The activity of alkaline amylase in mutant *B. subtilis* 168 mut-16# strain was 1.34-fold greater than that in the wild-type, and the highest specific production rate was improved from 1.31 U/(mg·h) in the wild-type strain to 1.57 U/(mg·h) in the mutant strain. Meanwhile, the growth of *B. subtilis* was significantly enhanced by ARTP mutagenesis. When the agitation speed was 550 rpm, the highest activity of recombinant alkaline amylase was 1.16- and 1.25-fold of the activities at 450 and 650 rpm, respectively. When the concentration of soluble starch and soy peptone in the initial fermentation medium was doubled, alkaline amylase activity was increased 1.29-fold. Feeding hydrolyzed starch and soy peptone mixture or glucose significantly improved cell growth, but inhibited the alkaline amylase production in *B. subtilis* 168 mut-16#. The highest alkaline amylase activity by feeding hydrolyzed starch reached 591.4 U/mL, which was 1.51-fold the activity by feeding hydrolyzed starch and soy peptone mixture. Single pulse feeding-based batch feeding at 10 h favored the production of alkaline amylase in *B. subtilis* 168 mut-16#.

**Conclusion:**

The results indicated that this novel ARTP mutagenesis-screening method could significantly improve the yield of recombinant proteins in *B. subtilis*. Meanwhile, fermentation optimization strategies efficiently promoted expression of recombinant alkaline amylase in *B. subtilis* 168 mut-16#. These findings have great potential for facilitating the industrial-scale production of alkaline amylase and other enzymes, using *B. subtilis* cultures as microbial cell factories.

## Background

Amylases (EC 3.2.1) are important industrial enzymes, one of which is alkaline amylase, which is stable under alkaline conditions. Alkaline amylase has significant potential for applications in the textile, paper, and detergent industries. Alkaline amylase is mainly present in alkalophilic microorganisms (e.g., *Bacillus licheniformis* and *Bacillus* sp.) [1–3]. Many alkaline amylases have been heterologously expressed in recombinant hosts to improve their yield and optimize their properties [4, 5]. Murakami et al. heterologously expressed alkaline amylase from *B. halodurans* MS-2-5 in recombinant *Escherichia coli*, and under optimized cultivation conditions, the amylase yield increased 104-fold compared with yield from the wild-type strain [4].

*B. subtilis*, a gram-positive bacterial strain, is an important industrial microorganism with a clear genetic background, is generally recognized as safe, has a superior secretion level, and is applicable for large-scale industrial products [6, 7]. *B. subtilis* is generally used to overexpress industrial enzymes (e.g., aminopeptidase, amylase, nattokinase, and protease) [6, 8–10]. Ploss et al. overproduced the industrially relevant amylase AmyM from *Geobacillus stearothermophilus* in *B. subtilis* 168 based on the secretion stress response [11]. There have been many strategies used to improve the expression of recombinant proteins in *B. subtilis*, including mutagenesis, screening highly efficient expression systems, strong promoters, peptides with high secretion level, and fermentation optimization [7, 9, 12–14].

ARTP (atmospheric and room temperature plasma) has been used as a novel mutagenesis technology in mutagenesis breeding of microorganisms (e.g., bacteria, actinomycetes, and fungi) to improve the yield of industrial products [9, 15]. In our previous work, a *B. subtilis* WB600 mutant with a high yield of recombinant alkaline amylase was screened by ARTP mutagenesis technology and a high-throughput screening technique (HTS) [9]. Fed-batch culture is frequently applied to improve product yield and productivity in industrial microbial processes by preventing catabolite repression and substrate inhibition [13]. Park et al. analyzed the effect of controlling amino acid composition in a fed-batch culture on amylase production in recombinant *B. brevis*, the maximum yield of which was obtained by controlling high asparagine and isoleucine concentrations and low other amino acids concentrations, increased from 5.14 kU/mL to 12.01 kU/mL [16].

In our previous work, *B. subtilis* WB600 without any recombinant plasmids was induced by ARTP mutagenesis, which increased the difficulty of high-throughput screening because of the low efficiency of recombinant plasmid injection into *B. subtilis* [9]. In this work, *B. subtilis* 168 with recombinant plasmids was induced by ARTP mutagenesis. This strategy avoided the recombinant plasmid by injecting a *B. subtilis* mutant library after mutagenesis, which significantly improved the efficiency of mutagenesis and screening of *B. subtilis* mutants with a high expression level of recombinant proteins. The yield and specific production rate of recombinant alkaline amylase and the growth behavior of the mutant were determined and characterized. Moreover, fermentation optimization strategy was used to improve the production yield of recombinant alkaline amylase in *B. subtilis* mutant in a 3-L fermenter.

## Results and discussion

### ARTP mutagenesis and high throughput screening (HTS)

**Fig. 1:**
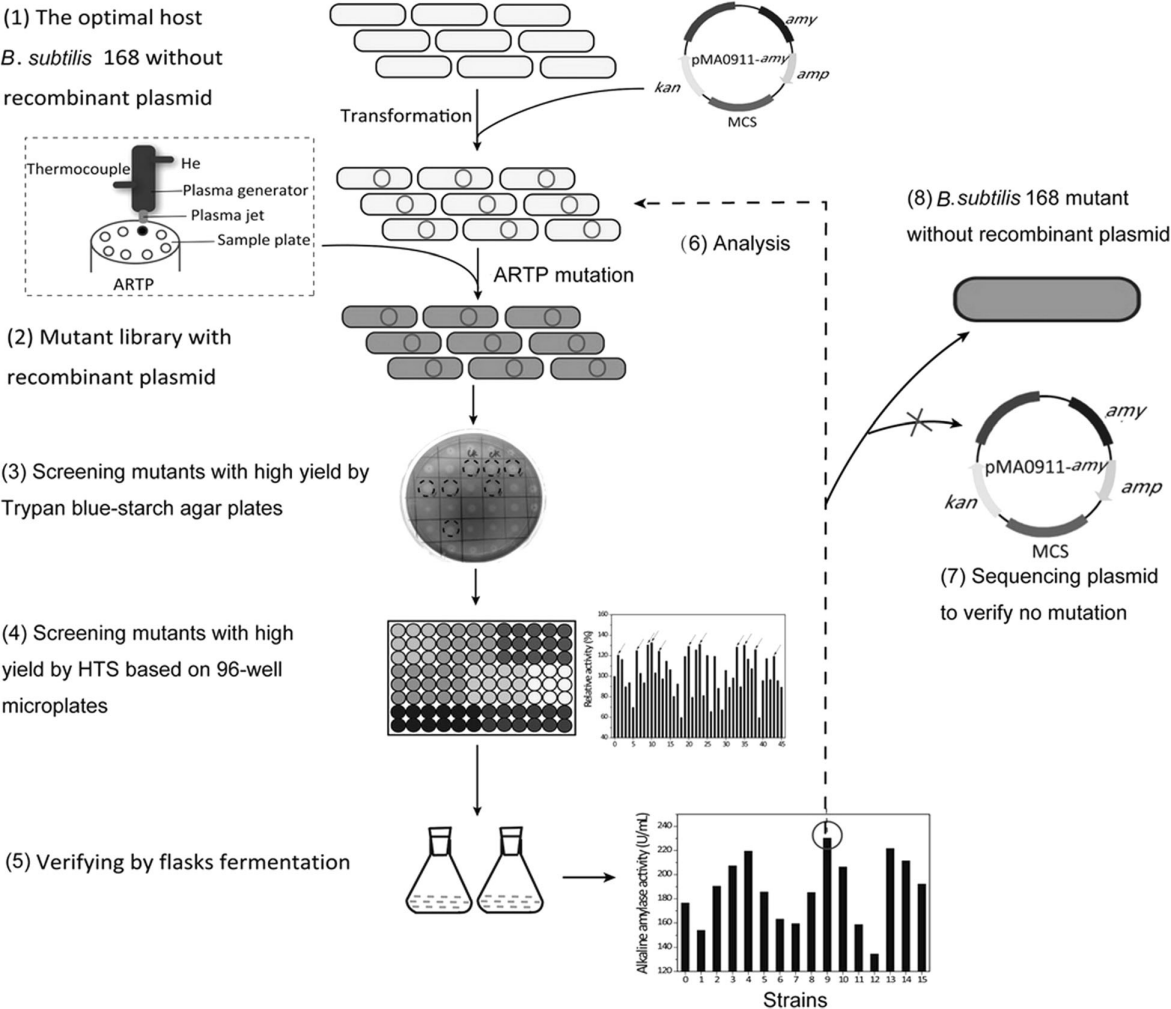
Novel ARTP and HTS operational procedures for the mutation and screening of *B. subtilis*

A novel ARTP mutagenesis and screening method was used to mutate *B. subtilis* 168 to improve the expression of recombinant alkaline amylase (Fig. 1). A mutant library of *B. subtilis* 168 harboring recombinant plasmids was successfully established by ARTP mutagenesis. Recombinant *B. subtilis* 168 mutants in this library were effectively screened by trypan blue-starch agar plates and 96-well microplates. Based on HTS screening, twenty recombinant *B. subtilis* 168 mutants with high expression of alkaline amylase were selected for shake-flask fermentation. Among the strains analyzed, the yield of alkaline amylase in *B. subtilis* 168 mut-16# was the greatest.

**Fig. 2:**
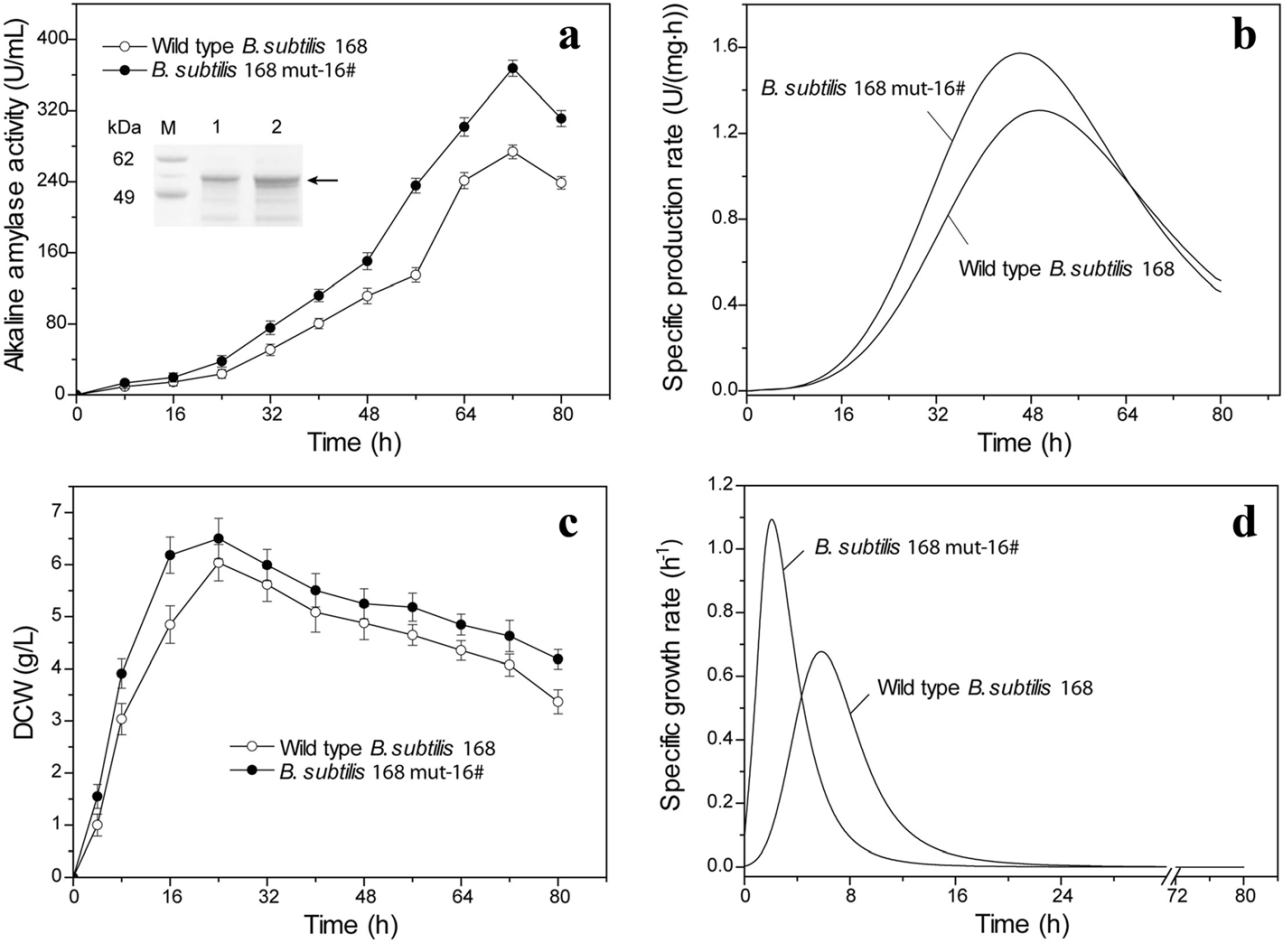
Comparison of *B. subtilis* mutants with the wild type strain for alkaline amylase production and strain growth. **a** alkaline amylase production. The inner is SDS-PAGE analysis. Arrow: alkaline amylase, 1: Wild type *B. subtilis* 168, 2: *B. subtilis* 168 mut-16#, M: standard protein marker. **b** specific production rate. **c** DCW. **d** specific growth rate

As shown in Fig. 2a, during shake-flask fermentation, the activity of alkaline amylase in *B. subtilis* 168 mut-16# reached 367.5 U/mL at 72 h, which was 1.34-fold the activity observed in the wild-type strain. In addition to increased activity, the concentration of recombinant alkaline amylase of *B. subtilis* 168 mut-16# was higher compared to the concentration observed for the wild-type strain. This indicated that ARTP mutagenesis strongly promoted extracellular production of recombinant alkaline amylase in *B. subtilis* 168. As shown in Fig. 2b, the highest specific production rate of alkaline amylase in *B. subtilis* 168 mut-16# was improved from 1.31 U/(mg·h) for wild-type strain to 1.57 U/(mg·h) in the mutant strain. Additionally, the phase of highest specific production rate of alkaline amylase in *B. subtilis* 168 mut-16# moved up by 4 h compared with the that of the wild-type strain. This indicated that alkaline amylase production capacity in *B. subtilis* 168 mut-16# was enhanced by ARTP mutagenesis. ARTP mutagenesis could introduce the sublethal damages to organisms, such as DNA damage, permeability change, and protein denaturation [15, 17], which might be the main reason that alkaline amylase expression level in *B. subtilis* was significantly enhanced. DCW of *B. subtilis* 168 mut-16# was quickly increased in the initial fermentation phase (Fig. 2c). DCW of *B. subtilis* 168 mut-16# reached its peak of 6.5 g/L at 24 h; however, the peak of the wild-type strain DCW was only 6.0 g/L. The highest specific growth rate of *B. subtilis* 168 mut-16# was 1.12 h^−1^, which was 1.62-fold greater than the growth rate of the wild-type strain. The phase of the highest specific growth rate of *B. subtilis* 168 mut-16# was 1.6 h, which was lower than that (5.7 h) of the wild-type strain. This indicated that *B. subtilis* growth was significantly enhanced by ARTP mutagenesis. Meanwhile, Fang et al. also found that the ARTP, as an effective mutagenesis tool, could significantly improve the growth rate of *Spirulina platensis* FACHB 904 [18].

A recombinant plasmid containing the alkaline amylase gene in *B. subtilis* 168 mut-16# was obtained and sequenced, and the results showed that the plasmid had no mutations (data not shown). The genetic stability of *B. subtilis* 168 mut-16# was also evaluated by continuous subcultivation. The results suggest that this strain exhibited good genetic stability (data not shown). In previous studies, *Streptomyces albulus* A-29 and *Enterobacter cloacae* (MU-1) mutants also exhibited high genetic stability after ARTP mutagenesis [19, 20]; this indicates that ARTP mutagenesis is a promising tool for generating genetically stable mutants.

### Effect of agitation speed on alkaline amylase production in *B. subtilis* 168 mut-16#

**Fig. 3:**
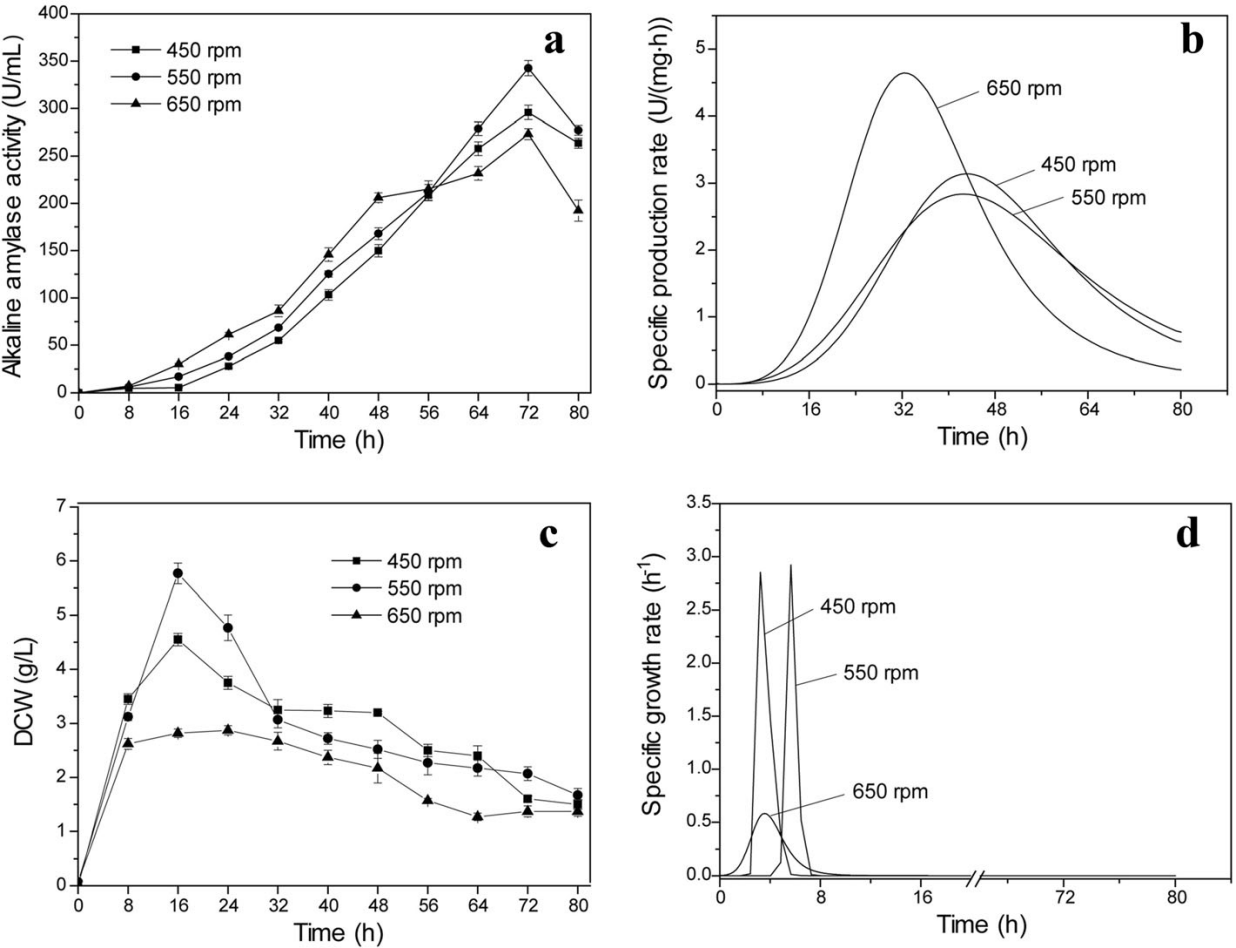
Effect of agitation speed on alkaline amylase production and growth of *B. subtilis*. **a** alkaline amylase production. **b** specific production rate. **c** DCW. **d** specific growth rate

Agitation speed is important for microorganism growth and synthesis of chemical products during fermentation. During aerobic fermentation, microorganisms require oxygen for growth and synthesis of chemical products. Dissolved oxygen concentration can affect microorganism metabolism and product yield. Agitation speed is a key factor affecting dissolved oxygen in the fermenter. The effect of different agitation speeds (450, 550, and 650 rpm) on *B. subtilis* 168 mut-16# growth and alkaline amylase production was examined in this study (Fig. 3). As shown in Fig. 3a, when the agitation speed was 550 rpm, the highest recombinant alkaline amylase activity reached 342.5 U/mL at 72 h, which was approximately 1.2- and 1.3-fold the activity compared to growth at 450 and 650 rpm, respectively. Range of specific production rate of alkaline amylase by *B. subtilis* 168 mut-16# at 550 rpm was wider than the ranges observed at other speeds, indicating that *B. subtilis* 168 mut-16# could efficiently produce alkaline amylase at 550 rpm for a prolonged period. At 650 rpm, the specific production rate of alkaline amylase production in *B. subtilis* 168 mut-16# reached its peak (3.1 U/(mg·h)) at initial phases, and then the rate quickly decreased. This suggested that high agitation speed favored recombinant enzyme production in *B. subtilis* 168 mut-16# at initial phases, but not in the late fermentation phase.

The DCW of *B. subtilis* 168 mut-16# was highest when grown at 550 rpm: 5.7 g/L (Fig. 3c). Meanwhile, the highest specific growth rate of *B. subtilis* 168 mut-16# was the highest at 550 rpm, indicating that these conditions favored quick growth of this strain. High agitation speed (650 rpm) favored quick growth of *B. subtilis* 168 mut-16# at initial phases, but the culture quickly reached stationary phase. These results suggested that high agitation speed promoted faster growth of *B. subtilis* 168 mut-16# at initial phases, but the higher shear force may also negatively affect growth at later phases.

### Effect of different soluble starch/soy peptone concentrations on alkaline amylase production by *B. subtilis* 168 mut-16#

**Fig. 4:**
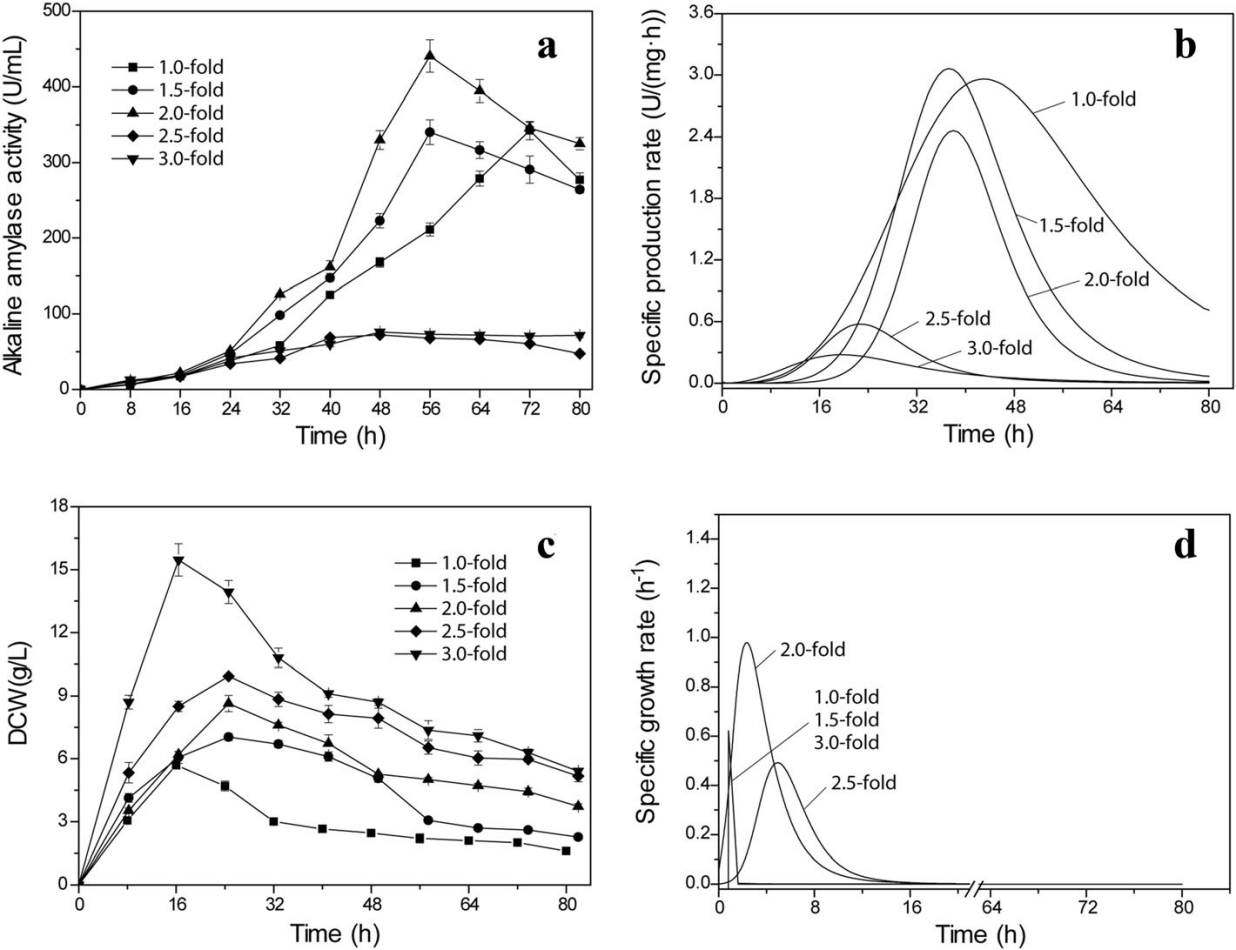
Effect of different soluble starch/soy peptone (SSSP) concentrations on alkaline amylase production and growth of *B. subtilis*. **a** alkaline amylase production. **b** specific production rate. **c** DCW. **d** specific production rate. Soluble starch and soy peptone ratios were consistent

Mediums could affect the promoter activity, and homogenous populations of cells with highly productive is necessary for the production of secretory enzymes in industrial-scale fermentations [11, 21]. High cell concentration is a novel strategy for improving the yiled of recombinant enzymes in *B. subtilis* [22]. Strategies improving *B. subtilis* cell concentration mainly included medium optimization, fed-batch without feedback control (e.g., pulse feeding), fed-batch with feedback control (e.g., indirect feedback control strategy) [13, 23]. Dual feeding with defined media is important for producing recombinant proteins in *B. subtilis* [24]. In this study, the cell concentration of *B. subtilis* was improved by increasing the main carbon and nitrogen source (soluble starch and soy peptone) concentrations in the initial fermentation medium to enhance the yield of recombinant alkaline amylase production. As shown in Fig. 4a, the recombinant alkaline amylase yield was significantly improved by increasing the soluble starch/soy peptone (SSSP) concentration. When the SSSP concentration of the initial medium was doubled, the activity of alkaline amylase in *B. subtilis* 168 mut-16# reached 440.7 U/mL at 56 h, which was 1.3-fold greater than the activity in the initial medium. However, when SSSP concentration was increased more than 2-fold compared to the initial medium, activity of recombinant alkaline amylase was significantly decreased to approximately 70.0 U/mL. When SSSP concentration was 1.5-fold that of initial medium, the highest specific production rate was 3.1 U/(mg·h) (Fig. 4b). However, when SSSP concentration was more than double that in the initial medium, the highest specific production rate was significantly decreased. These results indicated that high initial carbon and nitrogen source concentrations were not preferred for producing recombinant alkaline amylase in *B. subtilis*. As shown in Fig. 4c and d, the cell concentration of recombinant *B. subtilis* 168 mut-16# was significantly improved by increasing the SSSP concentration. When the SSSP concentration was 1.0-, 1.5-, 2.0-, 2.5-, and 3.0-fold that of initial medium, the highest DCWs obtained were 5.7, 7.0, 8.6, 9.9, and 15.5 mg/L, respectively. When SSSP concentration was 2.0-fold higher than that in the initial medium, the peak specific growth rate of *B. subtilis* 168 mut-16# was the highest. This indicated that high SSSP concentration in the initial medium was preferable for cell growth of *B. subtilis* 168 mut-16#. In conclusion, high SSSP concentration was preferred for recombinant enzyme production and cell growth; however, excessively high concentrations could promote quick cell growth, but inhibit recombinant enzyme production. Yao et al. found that high initial medium concentrations (e.g., glucose) could result in excessive cell growth, but poorly enhance the production level of target biomolecules in *B. subtilis* [25]. Kwon et al. investigated effect of high cell density culture on nattokinase production in *B. subtilis* by pH-stat fed-batch, and found that the cell concentration reached highest at a ratio of 0.2 g glucose/g peptone, but the nattokinase activity was highest at a ratio of 0.33 g glucose/g peptone [22]. It was found that the presence of subpopulations of high-level and low-level proteins producing cells when cells were cultured in LB medium [11, 26, 27]. In this work, when SSSP concentration was excessively high, quick growth cells might include many low-level proteins producing cells.

### Effect of different feeding compositions on alkaline amylase production in *B. subtilis* 168 mut-16#

Feeding with appropriate nutrients favors the optimal expression of transcription promoters and effective secretion of heterologous proteins by *B. subtilis* [28]. Media comprising the required nutrients for supporting strain growth and preventing the inhibition of growth were generally fed to the bacteria to improve protein yield [13]. Batch feeding of carbon sources with catabolite-repressing could significantly improve the expression level of proteins (e.g., amylase) with carbon catabolite repression [29]. Based on the above optimum conditions, a fed-batch strategy was used to improve the yield of alkaline amylase in *B. subtilis* 168 mut-16# in this study. The different feeding compositions included glucose, hydrolyzed starch, and a concentrated mixture of hydrolyzed starch and soy peptone. Following application of these carbon sources, the pH of the fermentation medium increased, and fed-batch cultures controlled by pH change have been used for producing recombinant proteins and chemical products [28, 30, 31]. Based on changes in pH, the effect of different feeding compositions on the production of recombinant alkaline amylase in *B. subtilis* 168 mut-16# was investigated.

**Fig. 5:**
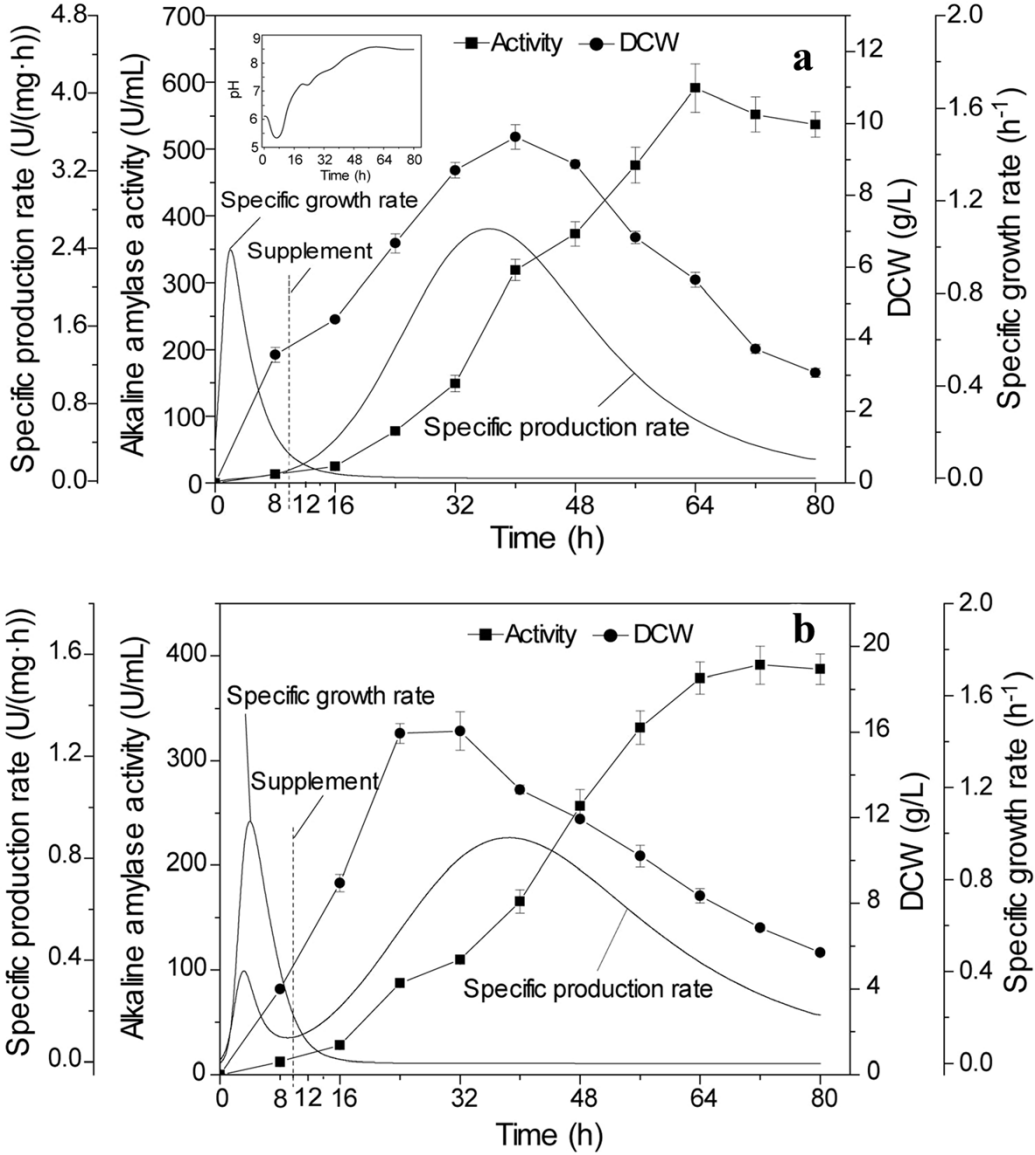
Effect of different feeding compositions on alkaline amylase production and growth of *B. subtilis*. **a** Effect of feeding hydrolyzed starch on alkaline amylase production and growth of *B. subtilis*. The inner: pH change curve during fermentation. **b** Effect of feeding a combination of hydrolyzed starch and soy peptone on alkaline amylase production and growth of *B. subtilis*

Glucose is usually applied as a growth-limiting substrate [13]. However, in this study, when solely glucose was used for feeding, recombinant alkaline amylase was not expressed by *B. subtilis* 168 mut-16# (data not shown), indicating that glucose as a fast-utilized carbon source inhibited the expression of recombinant alkaline amylase in this strain. Meanwhile, Zhang et al. also found that glucose feeding had a negative effect on the expression of penicillin G acylase from *Alcaligenes faecalis* in *B. subtilis* [32]. Soluble starch was utilized relatively slowly to avoid excessive glucose concentration to inhibit expression level of recombinant proteins in *B. subtilis*. However, the solubility of soluble starch is low (<50 g/L), and could result in oxygen transfer by poor mixing [32]. The solubility and fluidity of hydrolyzed starch can be significantly improved, which can prevent high glucose concentrations in the medium. The activity of alkaline amylase upon adding hydrolyzed starch was the highest and reached 591.4 U/mL at 64 h, which was 1.3-fold greater than batch fermentation (Fig. 5a). The highest specific alkaline amylase production rate on adding hydrolyzed starch reached 2.6 U/(mg·h), which was higher than the rate obtained by batch fermentation. Meanwhile, the highest DCW of *B. subtilis* 168 mut-16# was 9.6 g/L at 40 h, which was 1.4-fold the DCW obtained by batch fermentation. These results indicated that adding hydrolyzed starch promoted cell growth and production of recombinant alkaline amylase in *B. subtilis* 168 mut-16#. To avoid a lack of nitrogen, soy peptone was added to the media with hydrolyzed starch. As shown in Fig. 5b, adding a mixture of hydrolyzed starch and soy peptone significantly improved DCW of *B. subtilis* 168 mut-16#. The highest DCW reached 14.3 g/L, which was 2.0-fold the DCW obtained by batch culture. However, the highest alkaline amylase activity was only 391.1 U/mL, lower than the activity obtained by adding hydrolyzed starch. The highest specific alkaline amylase production rate (0.9 U/(mg·h)) was 36 % of the rate observed on adding hydrolyzed starch. This indicated that adding a nitrogen source (soy peptone) promoted the growth of *B. subtilis* 168 mut-16#, but inhibited the alkaline amylase yield. Cho et al. also found that although supplementation of yeast extract significantly improved *B. subtilis* cell growth, recombinant nattokinase activity was low [33].

### Effect of different feeding methods and times on alkaline amylase production in *B. subtilis* 168 mut-16#

**Fig. 6:**
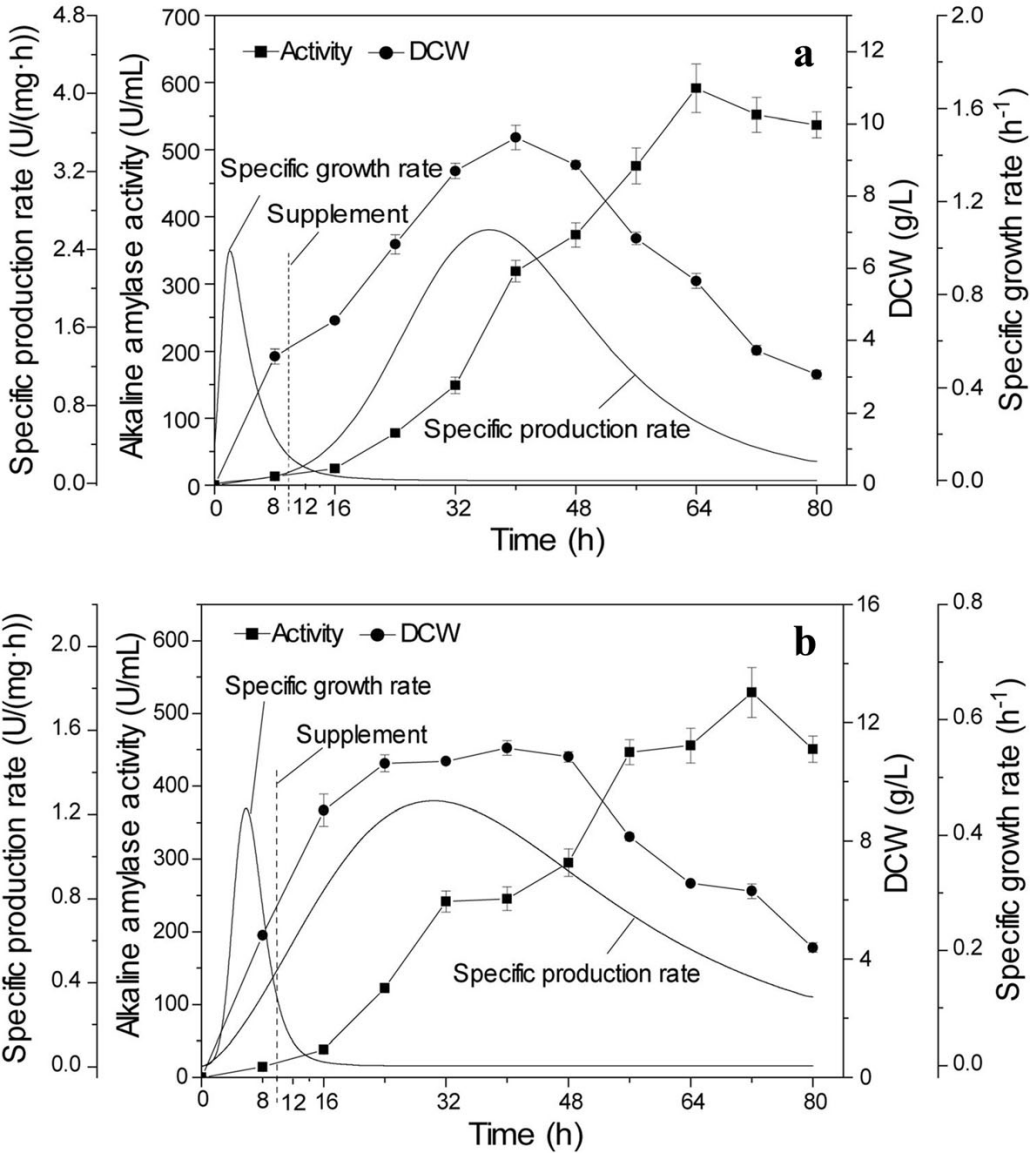
Effect of different feeding methods on alkaline amylase production and growth of *B. subtilis*. **a** Effect of fed-batch with single feeding on alkaline amylase production and growth of *B. subtilis*. b Effect of fed-batch with continuous feeding on alkaline amylase production and growth of *B. subtilis*

Constant feed flow rate-based batch feeding prevented the inhibitory effect of high substrate concentration on cell growth compared with single pulse feeding-based batch feeding [13]. As shown in Fig. 6, the effects of single pulse feeding versus constant feed flow rate-based batch feeding on alkaline amylase production and cell growth were compared and studied, while the quantity of hydrolyzed starch fed and initial feeding time were held constant. When hydrolyzed starch was continuously fed at 1.0 mL/min at 10 h, the highest yield of alkaline amylase was 528.7 U/mL, lower than the yield obtained from single pulse feeding-based fed-batch (Fig. 6b). The highest specific production rate from constant feed flow rate-based fed-batch was only 50 % of the rate obtained from single pulse feeding-based fed-batch. However, the highest DCW of a constant feed flow rate-based fed-batch was 11.1 g/L, higher than the DCW obtained from single pulse feeding-based fed-batch. These results indicated that constant feed flow rate-based feeding promoted cell growth in *B. subtilis* 168 mut-16#, but inhibited the expression of recombinant alkaline amylase. High cell densities might be generated with stressing the cells at initial stage for constant feed flow rate-based feeding in this work, which might include many low-producing cells [11].

**Fig. 7:**
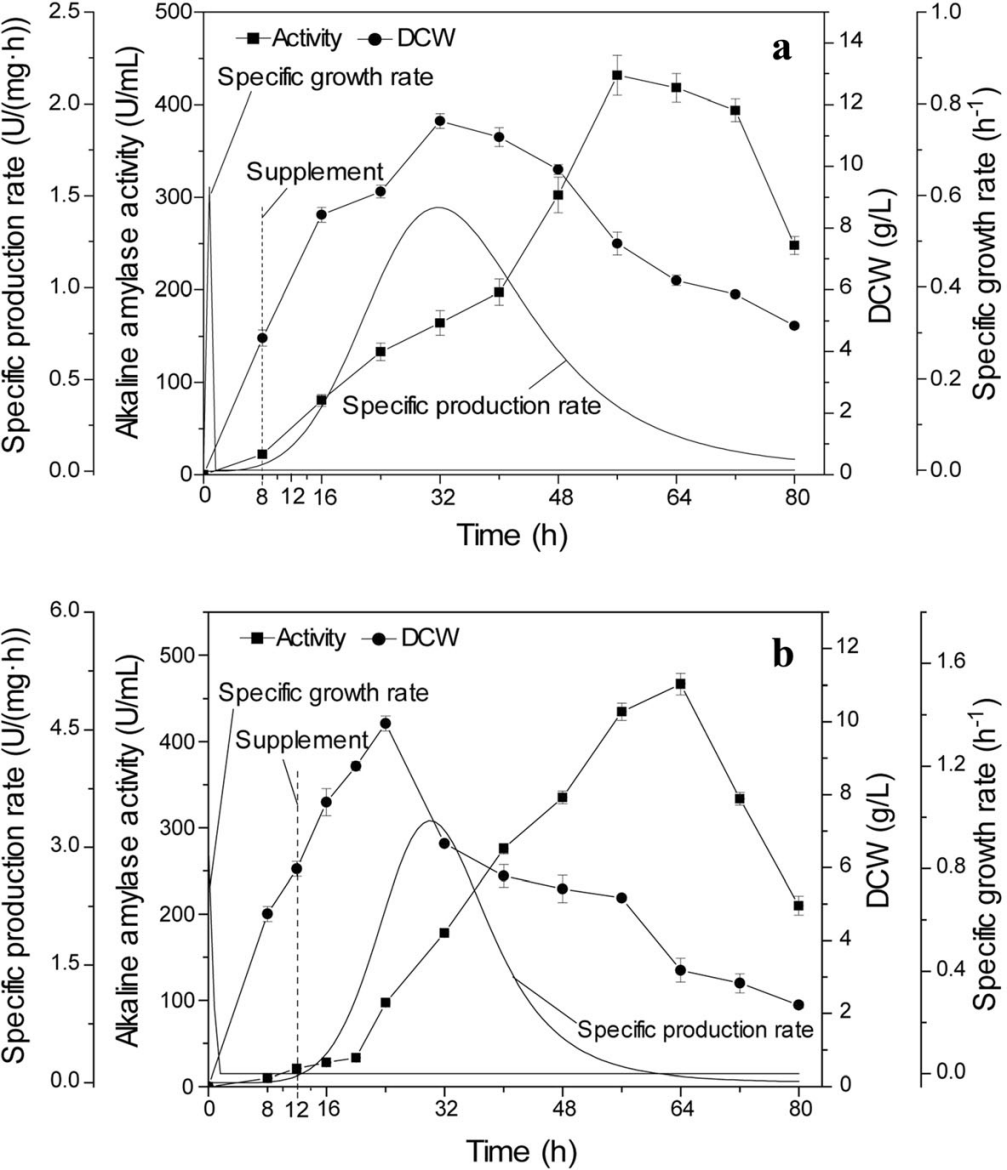
Effect of different feeding times on alkaline amylase production and growth of *B. subtilis*. Effect of feeding at (**a**) 8 h and (**b**) 12 h on alkaline amylase production and growth of *B. subtilis*

As shown in Fig. 7, the effect of feeding time on alkaline amylase production and cell growth was investigated and analyzed. When hydrolyzed starch was fed at 8 and 12 h, the highest enzyme activities, 431.5 and 466.7 U/mL, respectively, were lower than the activity obtained upon feeding at 10 h. The highest specific production rates upon feeding at 8 and 12 h were 1.4 and 3.3 U/(mg·h), respectively. The highest DCWs from feeding at 8 and 12 h were 11.5 and 8.4 g/L, respectively. These results indicated that feeding too early or too late could result in outgrowth or hypotrophy and inhibit alkaline amylase production in *B. subtilis* 168 mut-16#. Different growth stages with high cell densities could significantly influence high-yield fermentations for protein production in *B. subtilis* [11].

## Conclusion

*B. subtilis* 168 mut-16#, the mutant with the highest yield of alkaline amylase, was obtained by novel ARTP mutagenesis-screening method. The cell growth and recombinant alkaline amylase production capacity in *B. subtilis* 168 mut-16# were significantly enhanced by ARTP mutagenesis in this study. An agitation speed of 550 rpm favored alkaline amylase production by *B. subtilis* 168 mut-16# and resulted in fast growth. A high concentration of soluble starch and soy peptone was preferred for cell growth and recombinant enzyme production by *B. subtilis* 168, while excessively higher concentrations promoted faster cell growth but inhibited recombinant enzyme production. Feeding hydrolyzed starch promoted the growth and recombinant alkaline amylase production by *B. subtilis* 168 mut-16#. Glucose, a quickly utilized carbon source, inhibited recombinant alkaline amylase expression in this *B. subtilis* strain. Feeding with a nitrogen source (soy peptone) promoted the growth of *B. subtilis* 168 mut-16#, but inhibited the yield of alkaline amylase. Single pulse feeding-based fed-batch promoted the expression of recombinant alkaline amylase in *B. subtilis* 168 mut-16#. Feeding too early or too late could result in outgrowth or hypotrophy and inhibit the production of alkaline amylase in *B. subtilis* 168 mut-16#. In the future, we will investigate the effect of integration of more rounds of ARTP mutagenesis with systems-level fermentation optimization including as many high-producing cells on high-level gene expression in *B. subtilis*.

## Methods

### Microorganisms and media

The wild-type strain *B. subtilis* 168 and the shuttle plasmid pMA0911-*amy* (containing an alkaline amylase gene) were maintained in our culture collection center. Luria-Bertani (LB) medium was used for the *B. subtilis* starter culture. The trypan blue-starch agar plate included 10.0 g/L soluble starch, 0.2 g/L trypan blue, and 100.0 μg/mL kanamycin. The initial fermentation medium included 10.0 g/L soluble starch, 5.0 g/L NaCl, 30.0 g/L soy peptone, 20.0 g/L soybean meal, and 100.0 μg/mL kanamycin.

### Culture conditions

The working volume of flask culture was 25 mL/250 mL. The *B. subtilis* starter culture was grown at 37 °C and 200 rpm for 10 h. The starter culture inoculum was 4.0 % when transferred into the fermentation medium in the 3-L fermenter. *B. subtilis* was cultured at 37 °C to produce recombinant alkaline amylase.

### ARTP mutagenesis and high throughput screening (HTS)

The experimental protocols for ARTP and HTS are shown in Fig. 1. Methods and materials used for ARTP mutagenesis were the same as the materials used in our previous study [9]. Trypan blue-starch agar was used to screen for *B. subtilis* with high amylase expression to improve the screening efficiency. Based on the size of the transparent rings, *B. subtilis* mutants with high alkaline amylase activity were selected, and were further screened by HTS.

### Screening and verification of *B. subtilis* mutants with high alkaline amylase activity

*B. subtilis* mutants with high yield of recombinant alkaline amylase were further verified and screened by flask fermentation. After HTS, *B. subtilis* mutants with a high yield of recombinant alkaline amylase were cultured in 250 mL shaker flasks with 25 mL fermentation medium at 200 rpm and 37 °C. After verification by shaker flask fermentation, the recombinant plasmid in *B. subtilis* mutants with the highest yield was obtained and sequenced to verify a lack of mutations. *B. subtilis* mutant genetic stability was examined by subculturing mutants for 20 generations [9].

### Analysis of amylase activity

One unit (U) of amylase was defined as the amount of enzyme required for catalyzing starch to release 1 μmol reducing sugar (glucose) per minute at 50 °C and pH 9.5 [9]. Amylase activity was determined by a modified DNS (3,5-dinitrosalicylic acid) method [9].

### Biomass assay

Dry Cell Weight (DCW) was determined to analyze *B. subtilis* cell concentration. The soybean meal was first sedimented by low-speed centrifugation (600 × *g*, 30 s). Then, *B. subtilis* was centrifuged at 12,000 × *g* for 5 min and washed with NaCl solution (0.9 %, w/v). Cells were dried at 105 °C for 2 h and weighed on an electronic balance.

### Fed-batch strategy

To study the effect of agitation speed on the production of alkaline amylase in recombinant *B. subtilis*, agitation speeds during 3-L fermentation were maintained at 450, 550, and 650 rpm. To study the effects of different soluble starch and soy peptone concentrations, soluble starch and soy peptone concentrations in the fermentation medium were 1.0-, 1.5-, 2.0-, 2.5-, and 3.0-fold of the initial concentration. To study the effect of different feeding compositions on alkaline amylase production and growth of *B. subtilis*, glucose, hydrolyzed starch, and hydrolyzed starch and soy peptone were fed after 10 h of culture. Soluble starch (200 mL 20 % (w/v)) was hydrolyzed by 0.04 g thermostable amylase (20,000 U/g) at 100 °C until the blue color of iodine and potassium iodide solution stabilized. The ratio of hydrolyzed starch to soy peptone used was 2:3. To study the effect of the feeding method on alkaline amylase production and growth of *B. subtilis*, fed-batch methods included single pulse feeding or constant feed flow rate-based feeding. The constant feed flow rate was 1.0 mL/min. Different feeding times of 8, 10, and 12 h were studied.

### Statistical analysis

Experiments were independently performed 3 times, and the data shown are the mean of these replicates. Errors are shown as the standard deviations (SD).
